# Enhanced Fouling Resistance and Antimicrobial Property of Ultrafiltration Membranes Via Polyelectrolyte-Assisted Silver Phosphate Nanoparticle Immobilization

**DOI:** 10.3390/membranes10100293

**Published:** 2020-10-17

**Authors:** Kunal Olimattel, Jared Church, Woo Hyoung Lee, Karin Y. Chumbimuni-Torres, Lei Zhai, A H M Anwar Sadmani

**Affiliations:** 1 Department of Civil, Environmental and Construction Engineering, University of Central Florida, Pegasus Drive, Orlando, FL 32816, USA; kunalolimattel@Knights.ucf.edu (K.O.); jkchurch@Knights.ucf.edu (J.C.); WooHyoung.Lee@ucf.edu (W.H.L.); 2 Department of Chemistry, University of Central Florida, 4000 Central Florida Blvd, Orlando, FL 32816, USA; Karin.ChumbimuniTorres@ucf.edu (K.Y.C.-T.); lzhai@ucf.edu (L.Z.); 3 NanoScience Technology Center and the Department of Chemistry, 12424 Research Parkway, Suite 400, Orlando, FL 32826, USA

**Keywords:** ultrafiltration membrane, polyelectrolyte, silver phosphate nanoparticles, functionalization, fouling

## Abstract

Ultrafiltration (UF) is a low-pressure membrane that yields higher permeate flux and saves significant operating costs compared to high-pressure membranes; however, studies addressing the combined improvement of anti-organic and biofouling properties of UF membranes are lacking. This study investigated the fouling resistance and antimicrobial property of a UF membrane via silver phosphate nanoparticle (AgPNP) embedded polyelectrolyte (PE) functionalization. Negatively charged polyacrylic acid (PAA) and positively charged polyallylamine hydrochloride (PAH) were deposited on the membrane using a fluidic layer-by-layer assembly technique. AgPNPs were immobilized within the crosslinked “bilayers” (BL) of PAH/PAA. The effectiveness of AgPNP immobilization was confirmed by microprofile measurements on membrane surfaces using a solid contact Ag micro-ion-selective electrode. Upon stable and uniform BL formation on the membrane surface, the permeate flux was governed by a combined effect of PAH/PAA-derived hydrophilicity and surface/pore coverage by the BLs “tightening” of the membrane. When fouled by a model organic foulant (humic acid), the functionalized membrane exhibited a lower flux decline and a greater flux recovery due to the electrostatic repulsion imparted by PAA when compared to the unmodified membrane. The functionalization rendered antimicrobial property, as indicated by fewer attachments of bacteria that initiate the formation of biofilms leading to biofouling.

## 1. Introduction

Membrane fouling may result from an accumulation of filtered organic and inorganic materials and biofilms on the membrane surface and within the pore walls, causing a loss of flux over time. The fouling may be temporary if flux can be recovered by cleaning the surface or backwashing. Otherwise, the membrane would be fouled “irreversibly”. Recently, considerable attention has been given to surface modification of membranes to tackle the issue of fouling [1,2,3,4,5,6,7]. Considering how the membrane surface serves as an “active layer” in the filtration process, the choice by researchers to explore surface modifications to achieve antifouling membranes has become apparent. Recent studies aimed at modifying membrane surfaces using nanoparticles (NPs) [8,9,10,11,12], thin-film nanocomposites (TFNs) [13,14], and biologically inspired membranes [15,16,17] have demonstrated promising improvements in antifouling performance. Among various membrane modification techniques, producing polyelectrolyte (PE) multilayers through layer-by-layer (LbL) molecular-level adsorption of polymers is a well-established methodology that results in conformal thin-film coatings with precisely tuned physical and chemical functionalities [18,19,20,21,22,23,24,25]. A sequential deposition of materials during the LbL technique facilitates intermolecular interactions including electrostatic interactions [26,27], hydrogen bondings [28,29,30,31], and metal complexation [32,33,34]. The LbL technique is often chosen because of its ability to generate chemically stable ultra-thin films and flexibility with respect to the coating thickness and surface charge [35,36]. These coatings have been shown to be super-hydrophilic, which is the main reason for these being used to coat the surface of the membrane [37,38].

The incorporation of engineered nanomaterials including carbon-based materials, metal oxides, and metals in polymeric membranes has been demonstrated as a promising way to mitigate membrane fouling [39,40,41,42,43]. Nanocomposite membranes embedded with silver nanoparticles (AgNPs) have shown enhanced anti-adhesive properties, reduced bacterial cell density on membrane surfaces [44,45], and improved anti-biofouling performance [44,45,46]. The antibacterial mechanism of AgNPs can be explained mainly in two ways. The first is through the release of Ag ions that bind with chemicals in the cell, which in turn disrupt vital metabolic processes, killing the bacterial cell. The second mechanism involves damaging the microbial cell wall through the action of reactive oxygen species (ROS) and subsequent osmotic collapse [45,47,48]. Nevertheless, the integration of AgNPs with polymeric membranes and determining the stability of the integrated particles under pressure-driven filtration remain challenging tasks.

When considering membrane functionalization aimed at fouling mitigation, most studies have demonstrated the improvement of a specific membrane property, which might have been compromised with the membrane’s solute retention capability. Very few studies [49,50] have addressed the combined improvement of anti-organic and biofouling properties, and there is a lack of studies on the altered mechanisms of fouling as a result of membrane modification, more specifically, in the case of low-pressure polymeric membranes. Firouzjaei et al. [50] incorporated graphene oxide and Ag-based nanomaterials into thin-film nanocomposite membranes to improve their antifouling and anti-biofouling properties; however, the performance evaluation was conducted under forward osmosis mode rather than under pressure-driven conditions. Accordingly, the stability of the embedded nanoparticles was not demonstrated under pressure-driven conditions. Another attempt to improve anti-biofouling and anti-organic fouling properties was focused on removing heavy metal ions by a nanofiltration (NF) membrane that was fabricated in the laboratory through interfacial polymerization (IP) between poly(piperazineamide) and trimesoyl chloride, followed by immobilization of AgNPs [49]. The approach of the current study was to functionalize an ultrafiltration (UF) membrane—a low-pressure (40–1000 kPa) membrane that yields higher permeate flux and saves significant operating costs compared to NF and reverse osmosis (RO) [51]—to equip it with enhanced anti-organic fouling and antimicrobial properties. Unlike the previous research, this study performed LbL membrane coating following a fluidic method using a cross-flow filtration setup to force the polyelectrolyte complex into the support structure and to ensure better uniformity of surface coverage of the coating [52,53].

This study investigated the mechanisms of fouling resistance and antimicrobial property of a UF membrane functionalized via polyelectrolyte (PE)-assisted silver phosphate nanoparticle (AgPNP) immobilization. PE multilayer films were first deposited on the membrane using a fluidic LbL technique and then crosslinked, followed by embedding of the AgPNPs within the PE coatings. It was hypothesized that such a functionally engineered membrane would exhibit enhanced fouling resistance due to the synergistic effects of surface charge enhancement, reduction of surface roughness, improved hydrophilicity, as well as antimicrobial properties. The technical challenges that are addressed in this study include the effectiveness of NP immobilization within the PE films (i.e., to ensure that the AgPNPs are not released from the membrane), the stability of the PE films with the integrated NPs, and the trade-off between membrane flux loss due to the conformal coating and flux enhancement as a result of hydrophilization of the membrane by the rich functional groups of PEs. For the first time in such a functionalization approach, microprofile measurements using a customized solid contact silver micro-ion-selective electrode on and near the PE modified membrane surfaces were conducted to evaluate the effectiveness of nanoparticle (i.e., the AgPNPs) immobilization within the PE layers.

## 2. Materials and Methods

### 2.1. Materials and Reagents

Polyacrylic acid (PAA) (Acros Organics^TM^, Fair Lawn, NJ, USA), 25 wt.% solution in water, and polyallylamine hydrochloride (PAH) (Alfa Aesar^TM^ Ward Hill, MA, USA), procured in solid powder form, were used to form the PE coatings. 1-ethyl-3-(3-dimethylamionopropyl) carbodiimide (EDAC) (Acros Organics^TM^, Fair Lawn, NJ, USA) was used as a crosslinking catalyst in the coating process. Silver acetate (AgC_2_H_3_O_2_) (99% pure analyte from Acros Organics^TM^, Fair Lawn, NJ, USA) and sodium hydrogen phosphate (Na_2_HPO_4_) (American Chemical Society (ACS) certified) were used to form AgPNP in the coatings. Humic acid (HA) sodium salt (50–60% as humic acid, Alfa Aesar, Ward Hill, MA, USA) was the model natural organic matter (NOM) [54] that was used in the fouling tests. The antimicrobial property of the membrane was probed using *E. coli* (ATCC 15597). Tryptic soy broth (TPB) and tryptic soy agar (TPA) (Becton & Dickinson, Sparks, Franklin Lakes, NJ, USA) were used to grow the bacterial cultures and as the plating medium, respectively.

### 2.2. Membrane Functionalization

**Figure 1:**
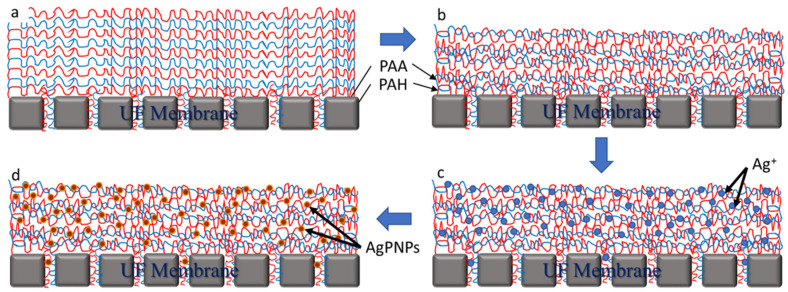
Schematic of the ultrafiltration (UF) membrane functionalization process sequence: (**a**) bilayer coating of virgin UF membrane with polyallylamine hydrochloride (PAH) and polyacrylic acid (PAA); (**b**) crosslinking of bilayers using 0.5% 1-ethyl-3-(3-dimethylamionopropyl) carbodiimide (EDAC); (**c**) Ag^+^ loading within the bilayers by soaking in 5 mM AgC_2_H_3_O_2_ solution; (**d**) formation of stable silver phosphate nanoparticle (AgPNPs) by soaking in 0.2 M Na_2_HPO_4_ solution.

A commercially available UF membrane (UA60, Trisep) was used in this study. This is a piperazine-based thin-film composite membrane that falls within the range between a “tight” UF and a “loose” NF membrane with a pore size distribution in the range of 1000 Daltons [55]. The deposition of negatively charged PAA and positively charged PAH coatings on membranes was performed by a fluidic method that was carried out by alternately circulating 0.01 M solutions of each of PAA and PAH through the cross-flow filtration cells for 2 min (loading time). This produced one PAH/PAA “bilayer” (BL) and this procedure was repeated till the PAH/PAA coatings of target thickness were obtained on the membrane. Deionized (DI) water was run for 30 s before changing solutions in order to flush out any remaining PAH or PAA from the previous cycle. The solutions were maintained at pH 3.5 for the duration of the coating process [56]. Crosslinking of the PAH/PAA bilayers was carried out by immersing the membrane in a 0.5% EDAC solution to improve the stability of the coatings [57]. This was followed by soaking of the membrane in a 5.0 mM silver acetate (AgC_2_H_3_O_2_) solution to infuse Ag ions into the BLs and then immersing it into a 0.2 M sodium hydrogen phosphate (Na_2_HPO_4_) solution to form stable AgPNPs (Figure 1). This resulted in PAH/PAA-assisted AgPNP immobilization on the UF membrane substrate. The virgin and functionalized membranes used in this study are listed in Table 1. 

### 2.3. Membrane Surface Characterization

The PAH/PAA and PAH/PAA/AgPNP layers on the membrane were examined using scanning electron microscopy (SEM) (Zeiss ULTRA-55 FEG, Oberkochen, Germany). Energy dispersive spectroscopy (EDS) was performed on the surface of the modified membrane to scan for the presence of AgPNPs [58]. Atomic force microscopy (AFM) was conducted using a Multimode SPM, NanoScope IIIA instrument (Veeco Instruments, Inc., Town of Oyster Bay, NY, USA). A cantilever tip, running in contact mode, was used to determine the surface roughness of the membrane. To quantify the membrane surface charge, zeta potential (ZP) measurements were carried out using an Anton-Paar Electrokinetic Analyzer at a range of pH values using KCl solution (10^−3^ M) in DI water as a background electrolyte, following a streaming potential method as described by Luxbacher [59]. Contact angle measurements via the sessile drop technique were taken using a Rame-Hart goniometer to determine the hydrophobicity/hydrophilicity of the virgin and modified membrane samples.

### 2.4. Silver Phosphate Nanoparticle (AgPNP) Stability Tests

The effectiveness of AgPNP immobilization within the PAH/PAA films was evaluated by monitoring the leaching of silver ions (Ag^+^) under different flow conditions. Accordingly, membrane samples loaded with AgPNP within the PAH/PAA BLs were compared with those with Ag^+^ infused within the BLs but not converted to AgPNPs (i.e., the immersion of the samples in Na_2_HPO_4_ was omitted). While PAA was deposited as the final layer during LbL deposition, one set of membrane samples was prepared with PAH as the topmost layer to verify if the charge imparted by the final layer had any impact on AgPNP stability.

**Figure 2:**
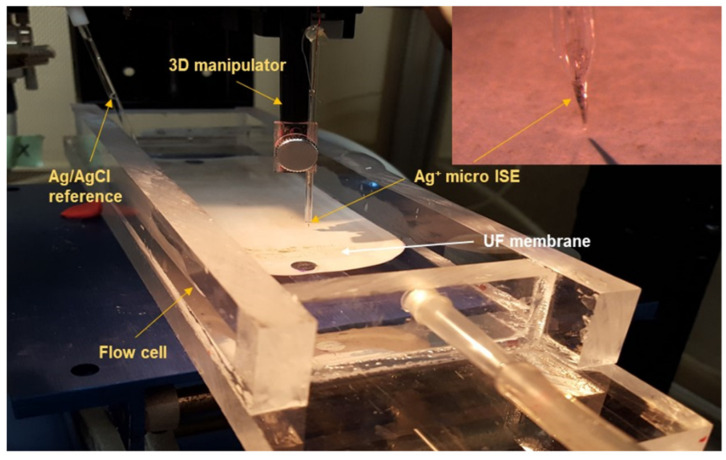
Silver (Ag) microprofile measurement setup (inset: closeup of Ag^+^ micro-ion-selective electrode (micro-ISE)).

Microprofiling of Ag^+^ concentrations on and near the membrane surface was conducted using a solid contact silver micro-ion-selective electrode (Ag^+^ micro-ISE), developed following protocols reported in recent studies [60,61,62]. The ion selective membrane composition responsible for Ag^+^ detection was prepared following another previous study [63]. The micro-ISE was calibrated using standard AgNO_3_ solutions with concentrations ranging from 10^−7^ M to 10^−2^ M. A commercial Ag/AgCl milli-electrode was used as an external reference electrode. The membrane samples were placed in a flow chamber (Figure 2) with a flowrate of 2 mL min^−1^ and allowed to equilibrate for 30 min. Microprofile measurements were taken from the bulk (2000 μm) to the membrane surface at 50 μm increments every 20 s. Three different locations on each membrane sample were examined to ensure uniformity, and duplicate profiles for each measurement were averaged to determine the concentrations. The samples that did not leach any Ag during the microprofile measurements were used to filter DI water for 48 h using the membrane filtration setup. Feed and permeate water samples were collected from the filtration run to determine Ag^+^ concentration using an inductively coupled plasma (ICP)-atomic absorption optical spectrometer (Optima 7300 DV Perkin-Elmer, Shelton, CT, USA) following Standard Method 3120 B [64]. The limit of detection of the instrument was 0.005 mg L^−1^.

### 2.5. Membrane Filtration Experiment

**Figure 3:**
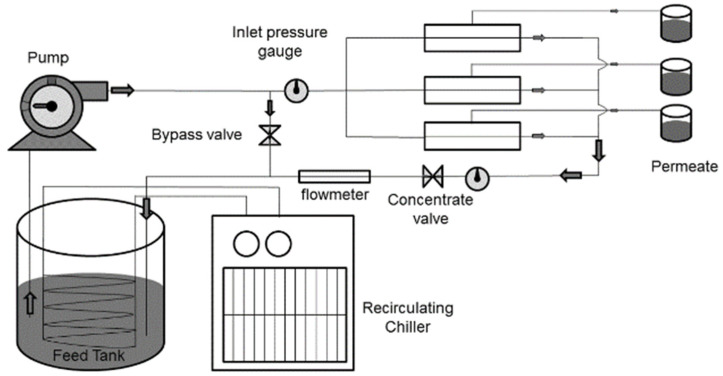
Schematic of bench-scale cross-flow membrane filtration setup.

The filtration experimental setup consisted of three parallelly connected cross-flow membrane cells (model: CF042A, Sterlitech, Kent, WA, USA), feedwater delivery pump, flow control valves, feed and concentrate pressure gauges, flow meters, chiller/heater (for temperature control), and storage tanks (Figure 3). To investigate the effect of the number of bilayer depositions on membrane permeability, DI water was filtered through the membranes. The membrane coupons were first compacted for 4 h and then the feedwater was circulated at a pressure of 40 psi. The pure water flux through the membrane was measured using Equation (1).

Flux (J) = V/(A × t) (1)

where V = volume of permeate (L), A = Active area of membrane coupon (m^2^), and t = time taken to collect permeate (h).

### 2.6. Fouling Protocol and Antifouling Performance Evaluation

Flat-sheet membrane coupons were compacted in the filtration cells with a 42 cm^2^ effective membrane area by circulating DI water for 4 h. The pressure was then adjusted to 80 psi and the pure water flux (F_1_) of the virgin membrane was determined by filtering DI water for 12 h. To determine antifouling performance of the modified membrane, the feedwater was spiked with 10 mg L^−1^ of commercially available humic acid (HA) and rejection experiments were conducted using virgin UF and PAH/PAA/AgPNP-functionalized UF (AgPNP-BL) membranes (Table 1). For the fouling run, the flux (F_HA_) was measured after circulating the HA-spiked feedwater for 12 h. The concentrations of HA in the feed and permeate samples were measured using ultraviolet absorbance at 254 nm wavelength (UVA_254_). After cleaning the system with DI water, the post fouling flux (F_2_) was determined through a DI water filtration run and Equations (2)–(5).

%Flux decline (FD) = (1 − F_HA_/F_1_) × 100% (2)

%Flux Recovery (FR) = (F_2_/F_1_) × 100% (3)

%Reversible flux decline (RFD) = [(F_2_ − F_HA_)/F_1_] × 100% (4)

%Irreversible flux decline (IrFD) = [(F_1_ − F_2_)/F_1_] × 100% (5)

The PAH/PAA/AgPNP-functionalized membrane was further evaluated for its antimicrobial property. Modified and virgin membrane coupons with 2 cm diameter were immersed in 20 mL of bacterial suspension inoculated with *E. coli*, which was previously cultured to log phase in TPB. The samples were continuously stirred at 200 rpm to avoid settling of the bacterial suspension. Samples were taken at the beginning of the test and after 24 h, serially diluted (10^0^; 10^−2^; 10^−4^; 10^−6^) and plated in triplicate on sterile TPA plates, and incubated at 35 °C for 24 h before counting of the bacterial colonies. Plate counts in terms of Colony Forming Units (CFU mL^−1^) were reported using the least dilute sample in the range of 30–300. The bacterial adhesion to the membrane surface was examined using SEM. Prior to scanning, the samples were prepared by immersing them in a 3% *v*/*v* glutaraldehyde solution for 5 h at 4 °C, then they were dehydrated in ethanol and dried in air.

A WITEC Alpha300 Raman Analyzer was utilized to compare the Raman spectra of surfaces of the virgin and modified membrane samples that were exposed to the *E. coli* suspension. Bacterial cells are made up of a number of proteins, lipids, nucleic acids, and saccharides. Raman spectroscopy produces unique responses based on the functional groups and their molecular vibration properties present in these cells [65,66]. Three different spots on each sample were selected and each spot was scanned 10 times. Thus, the average of 30 scans was used to generate the average spectral response for each sample.

## 3. Results and Discussion

### 3.1. UF Membrane Surface Properties Following PE-Assisted AgPNP Functionalization

**Figure 4:**
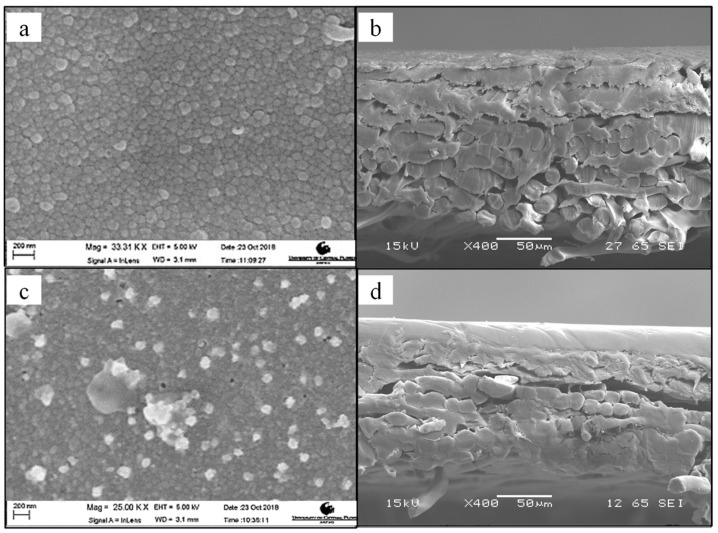
SEM images of a virgin membrane surface (**a**) and cross-section (**b**); 5 PAH/PAA BL-assisted AgPNP-immobilized UF membrane surface (**c**) and cross-section (**d**).

**Figure 5:**
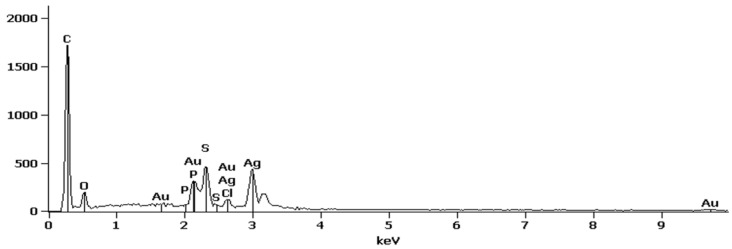
EDS spectra of 5 AgPNP-BL UF membrane surface.

**Figure 6:**
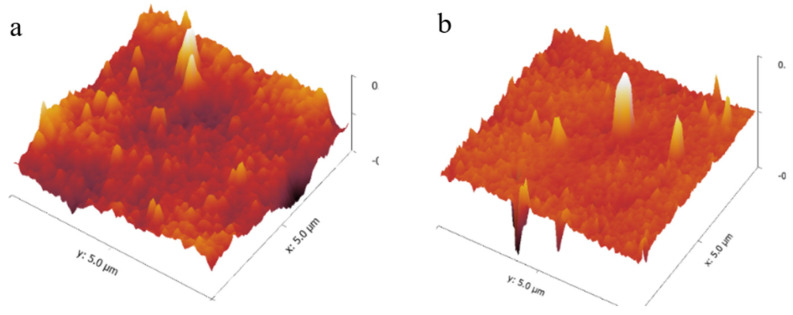
AFM surface profile of a virgin (**a**) and a modified (5 BLs) (**b**) UF membrane.

When comparing the SEM images (Figure 4a,c) of the virgin and modified UF membrane surfaces, the latter exhibited distinct bright specks, which were later confirmed as Ag by EDS scans (Figure 5). This indicated that the AgPNPs were successfully immobilized within the PAH/PAA BLs. The average loading of Ag, estimated from the EDS scans, was up to approximately 9% by weight on the 5 AgPNP-BL UF membrane. Ag ions show high affinity to carboxyl functional groups (—COOH) [67]. During membrane functionalization, the carboxylate ion groups (—COO^−^) from PAA likely bind the Ag^+^ within the bilayers for subsequent reduction to AgPNPs [68,69]. The pH of 3.5 used for the polyelectrolyte complex solutions in this study ensured that free carboxylic acid groups in the PAH/PAA bilayers were available for binding Ag^+^ [56,70]. The cross-sectional views (Figure 4b,d) show how a stable and uniform coating rendered a smoother surface on the modified membrane sample than on that of the virgin membrane. This is due to the inherent properties of the applied PEs, which form an ultra-thin coating on the surface of the membrane. AFM analysis demonstrated that the UF membrane functionalized with 5 AgPNP embedded PAH/PAA bilayers (AgPNP-BL) had a smoother surface with an average roughness (R_a_) of 10 nm and root mean square roughness (R_rms_) of 12.6 nm in comparison with that of the virgin membrane (R_a_ = 20.3 nm and R_rms_ = 26 nm) (Figure 6).

**Figure 7:**
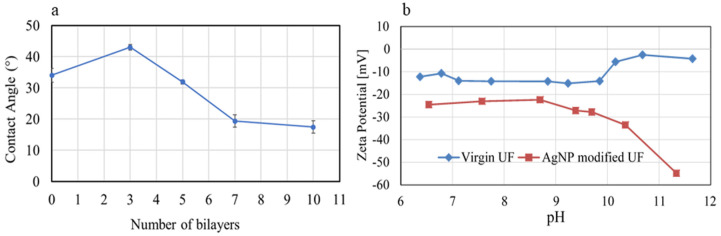
(**a**) Contact angles of virgin membrane (0 BL) and modified membranes as a function of the number of PAH/PAA BLs deposited (error bars represent standard deviation of 5 measurements); (**b**) Zeta potential of virgin and 5 AgPNP-BL membrane surfaces as a function of pH.

From contact angle measurements following different numbers of BL depositions, it appears that the UF membrane becomes more hydrophilic once stable BLs are formed (Figure 7a). Although 3 BLs resulted in a higher contact angle (CA = 43°) compared to that of the virgin UF membrane (34°), the contact angle began to decrease when the membrane was coated with 5 or more BLs. It required 5 BL depositions to achieve a stable and uniform coating (Figure 4), before which, the rather ‘loose’ PE molecules that might have accumulated on the membrane surface in a sporadic manner likely resulted in a rougher surface. An increase in surface roughness, as hypothesized to have resulted from 3 BL depositions in this case, may result in an increase in contact angle [71]. Once the membrane surface became smoother following 5 or more BL depositions, the functional groups of PAH and PAA imparted membrane hydrophilicity, as indicated by the gradual decrease in contact angle (Figure 7a).

The effect of membrane modification on surface charge is of importance, particularly when the organic fouling of membranes is considered [49,50]. Zeta potential (ZP) measurements exhibited that the modified membrane (5 AgPNP-BL) became markedly more negatively charged (over the range of pH values tested) compared to the virgin membrane (Figure 7b). The increased negative charge on the modified membrane is likely due to the deposited PAA as the top layer of the BLs, rendering the membrane’s antifouling property as a result of electrostatic repulsion between the membrane and foulants as discussed later in Section 3.4. The ZP was relatively steady over pH 7 to 8.5—the pH range typically practiced/maintained during water treatment.

### 3.2. AgPNP Stability Within PAH/PAA Layers

**Figure 8:**
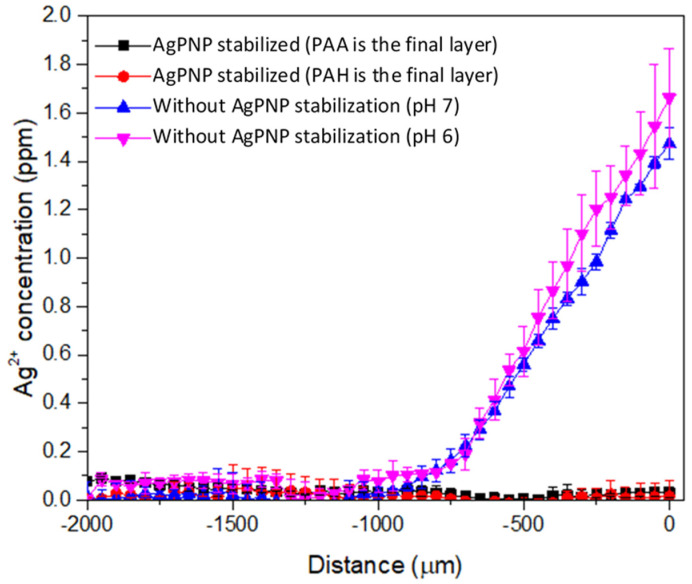
Microprofiling of Ag^+^ on and near functionalized UF membrane surfaces using Ag^+^ micro-ISE.

A custom needle type Ag^+^ microsensor was used to measure the concentration of Ag^+^ in situ near the membrane surface under flow conditions. With a very small tip diameter (20 µm), the sensor was applied to generate a concentration microprofile to provide mechanistic information on the release of Ag^+^ that cannot be obtained from bulk-scale measurements. In this study, the concentration gradients of Ag^+^ diffusion from a membrane with stabilized AgPNP was compared with that infused with Ag^+^, but not converted to stable AgPNP (Figure 8).

The Ag^+^ microprofiles demonstrated the release of Ag^+^ from the Ag^+^ infused membranes but not from the AgPNP decorated membranes. The linear increase in Ag^+^ concentration approaching the surface of the Ag^+^ infused membrane indicated that the release of Ag^+^ occurred through a diffusive process. Using Fick’s law, the Ag^+^ flux from the membrane at pH 7 and pH 6 was calculated to be 3.18 × 10^−7^ mg cm^−2^ s^−1^ and 4.07 × 10^−7^ mg cm^−2^ s^−1^, respectively. At this rate, the release of Ag^+^ into the environment could cause adverse impacts. The United States Environmental Protection Agency (USEPA) regulates silver to be less than 0.1 mg L^−1^ under the secondary drinking water standards of the Safe Drinking Water Act. Therefore, it is imperative that Ag^+^ is stabilized within the PAH/PAA layers of the membrane to avoid regulatory issues. For the samples with Ag^+^ converted to stable AgPNP, which were immobilized within the PAH/PAA bilayers, Ag^+^ measurements by the micro-ISE were below the detection limits (0.1 mg L^−1^) for all distances above the membrane surface, confirming the formation and immobilization of stable AgPNPs within the PAH/PAA layers. Furthermore, the final layer of the PAH/PAA BLs showed no influence on AgPNP immobilization since Ag^+^ concentrations were below the micro-ISE detection limit for both the cases when PAA or PAH was deposited as the final layer (Figure 8). When tested under a pressure-driven filtration condition, the Ag^+^ concentrations in the feed and permeate samples collected following 48 h of filtration were below the detection limit of ICP (0.005 mg L^−1^), indicating that the membrane samples with stable AgPNPs allowed negligible Ag leaching during pressure-driven filtration.

### 3.3. Effect of PAH/PAA BL Functionalization on UF Membrane Flux

**Figure 9:**
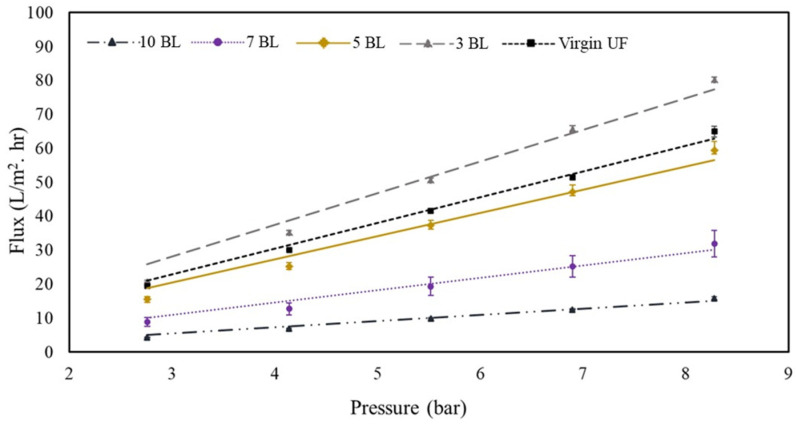
Effect of the number of PAH/PAA bilayers deposited on membrane permeate flux.

The permeate fluxes of the virgin and PAH/PAA modified UF membranes (PAH/PAA-UF) as a function of applied pressure are shown in Figure 9. The modified UF membranes are expected to offer NF-like performances as indicated by the overall flux trends [72]. The 3-BL membrane exhibited higher fluxes (pure water permeability, PWP = 8.9 L m^−2^ h^−1^ bar^−1^) compared to that by the virgin membrane (PWP = 7.6 L m^−2^ h^−1^ bar^−1^). The PWP by the virgin membrane is comparable to the that reported by the manufacturer (PWP = 7.9 L m^−2^ h^−1^ bar^−1^) [73]. While the hydrophilicity of the UF membrane was reduced due to 3 BL deposition (Figure 7a), the increased flux could be attributed to the surface roughness [74] caused by the sporadically deposited PAH and PAA molecules. It appears that stable films are effectively formed after the deposition of 3 BLs on the membrane substrate. This is in line with the findings of Mallwitz and Laschewsky [36], who developed a method to fabricate ultrathin freestanding PE films in the meshes of an electron microscopy grid. The initial films formed were so fragile that they could not be observed using optical microscopy. However, the films became stable after three cycles of coating and a slow drying process. The mechanical stability of the films was improved by adding more PE films and upon thermal crosslinking [36].

In the current study, as more stable and uniform BLs began to form on the surface, the PE-derived hydrophilicity was expected to result in increased permeate flux. However, the deposition of 5 BLs, despite increasing membrane hydrophilicity (CA~32° for the 5-BL membrane vs. ~34° for the unmodified membrane), resulted in slightly lower fluxes (PWP = 6.8 L m^−2^ h^−1^ bar^−1^) than the virgin membrane (PWP = 7.6 L m^−2^ h^−1^ bar^−1^). This is likely a combined effect of hydrophilicity and surface and pore coverage by the PAH/PAA BLs “tightening” the membrane. Since the fluidic method was applied in this study, it was expected that PAH and PAA would be forced into the support structure of the membrane in addition to coating the surface. As more BLs (7 and 10) were deposited, the membrane hydrophilicity was improved (Figure 7a), but permeate flux did not improve (PWP = 3.5 L m^−2^ h^−1^ bar^−1^ and 1.8 L m^−2^ h^−1^ bar^−1^ for 7 and 10 BLs, respectively). Park et al. [75] investigated PAH/PAA modification of commercial polysulfone substrates as an approach to improve desalination performance of RO membranes. They reported higher rejection (>99%) of NaCl, but markedly decreased permeate flux with the increase in the number of BLs deposited. The authors attributed this reduced flux performance to the formation of dense PAH/PAA films through crosslinking. This was further supported by the observation that the BL thickness increased exponentially as a function of BL numbers, particularly past 5 BLs [75]. In the current study, the number of BLs deposited was limited to five to ensure that the enhancement of membrane hydrophilicity and the resulting permeability were not offset by the hindrance from BL deposition.

### 3.4. Effect of PAH/PAA BLs on Organic Fouling Resistance and Flux Recovery

**Figure 10:**
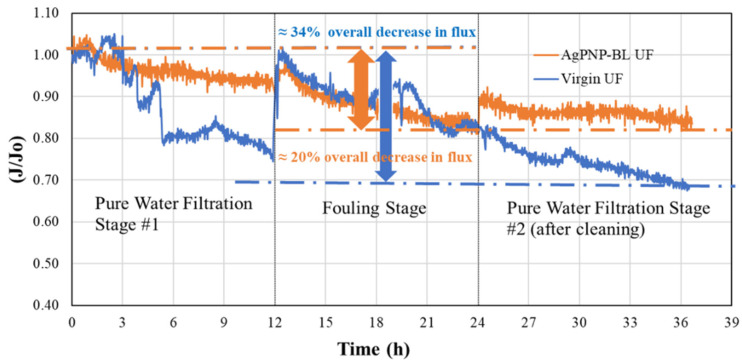
Normalized flux as a function of pressure during different stages of the fouling test.

The normalized flux (J/J_0_) values at different stages of the fouling test are shown in Figure 10. The virgin UF membrane showed a flux decline of 22.8 ± 8.8% in the presence of a model organic foulant, humic acid (HA), and 74.4 ± 16.1% of the original flux was recovered after cleaning. In contrast, the modified membrane (AgPNP-BL) showed a lower flux decline (13.8 ± 12%) and a greater flux recovery (86.2 ± 12%) compared to that of the virgin membrane. Hence, the overall flux decline by the functionalized UF membrane after 36 h was approximately 20%, whereas that for the unmodified commercial membrane was around 34%. The electrostatic repulsion resulting from increased surface negative charge upon PE functionalization, as indicated by the ZP measurements (Figure 7b), likely caused “loose” foulant layer(s), thereby contributing to the “reversibility” of flux. In comparison, when filtering HA-spiked feedwater, the unmodified membrane’s flux continued to decline even after cleaning (Figure 10).

### 3.5. Effect of AgPNP-Immobilized PAH/PAA BLs on UF Membrane Antimicrobial Property

**Figure 11:**
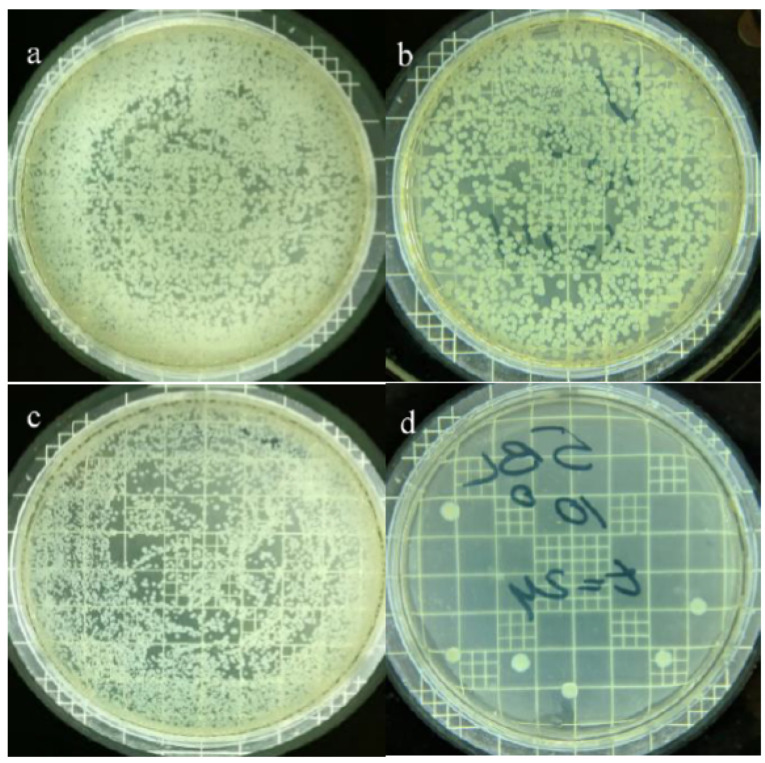
Plate counts (*E. coli*) on a virgin UF membrane at time (t) = 0 h (**a**) and at t = 24 h (**b**); plate counts on 5 AgPNP-BL UF membrane at time = 0 h (**c**) and at time = 24 h (**d**).

**Figure 12:**
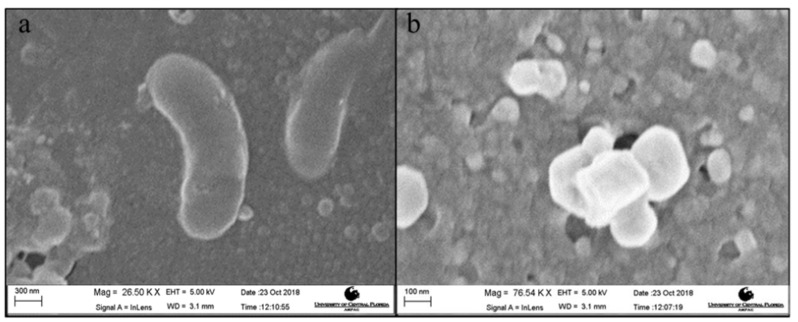
SEM images of membrane surface after 24 h exposure to *E. coli*: (**a**) virgin UF and (**b**) 5 AgPNP-BL UF membrane.

The virgin and modified UF membrane (AgPNP-BL) samples were exposed to a log phase bacterial suspension for 24 h. The average plate count for the AgPNP-loaded membrane was 8000 CFU mL^−1^, while that for the virgin UF membrane was 33,000 CFU mL^−1^ (Figure 11). Furthermore, the SEM image of the surface of the virgin membrane showed the presence of rod-shaped bacteria (Figure 12a), which were not observed on the AgPNP embedded membrane surface. The visible particles (Figure 12b) on the modified membrane resembled the immobilized AgPNPs that are shown in Figure 4c. This clearly demonstrates that the PAH/PAA BL assisted AgPNP immobilization imparted the antimicrobial property to the UF membrane, implying less activity [76] and attachment of microorganisms that initiate the multi-step process of biofilm formation on the membrane surface [77]. A previous study by Liu et al. [78], who tested a Ag-loaded chitosan/cellulose acetate blended membrane, suggested that functionalizing an anti-adhesive membrane surface with anti-microbial property was an effective way of providing anti-biofouling performance when compared to the approach of killing/inactivating the bacteria after attachment to the membrane surface.

**Figure 13:**
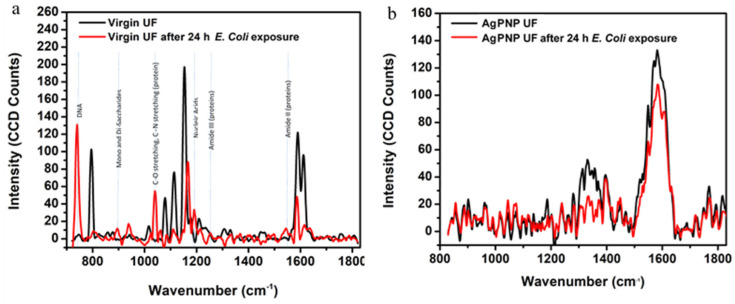
Raman spectra of membrane surface after 24 h exposure to *E. coli* for virgin (**a**) and 5 AgPNP-BL UF (**b**) membranes.

The Raman spectroscopic study of the membranes further demonstrates that the virgin UF membrane had bacterial biomass attached/deposited on the surface (Figure 13). Bio-molecular components (DNA, nucleic acids, mono and di-saccharides, and proteins) typical of *E. Coli* cells were identified on the surface of the virgin UF membrane sample that was exposed to the bacterial solution, confirming the accumulation of *E. Coli* in the absence of AgPNPs. In the case of the AgPNP-modified membrane, the Raman spectra did not exhibit much variation before or after *E. Coli* exposure, implying that the AgPNPs hinder the activity and attachment of *E. Coli* on the surface. This agrees with the plate counts from the static bacterial adhesion tests discussed above.

## 4. Conclusions

Ultrafiltration (UF) membranes were functionalized by immobilizing AgPNPs within polyelectrolyte (PAH/PAA) bilayers, rendering anti-organic fouling and antimicrobial properties to the membrane. Microprofile measurements on and near membrane surfaces using a customized solid contact Ag micro-ISE as well as post-filtration ICP analysis demonstrated that AgPNPs were effectively immobilized within the crosslinked PAH/PAA bilayers. This study reveals that the expected membrane flux enhancement due to PAH/PAA functionalization depends on a combined effect of PAH/PAA-derived hydrophilicity and surface and pore coverage by the stable BLs tightening the membrane. Hence, an optimum number of BLs (5 BLs in the current study) must be applied to ensure that the resulting permeability is not offset by the hindrance from BL deposition. When fouled by humic acid, the functionalized UF membrane exhibited a lower flux decline and a greater flux recovery when compared to the unmodified commercial membrane, likely due to the electrostatic repulsion imparted by PAA of the deposited BL. SEM studies, *E. Coli* plate counts, and Raman spectroscopic studies confirmed that the PAH/PAA-assisted AgPNP immobilization provided antimicrobial property to the UF membrane and caused fewer attachments of microorganisms that would have initiated the formation of biofilms ultimately leading to biofouling.

## Figures and Tables

**Table 1:** UF membranes tested and functionalized for different experiments in this study.

Membrane Tested/Functionalized	Membrane Description	No. of PAH/PAA Bilayers (BL) Deposited	AgPNP Immobilized?
Virgin UF	Commercial unmodified UF membrane	0	No
PAH/PAA-UF	UF membrane conformally coated with bilayers (BL) of PAH and PAA	1, 3, 5, 7, 10	No
AgPNP-BL	UF membrane functionalized by embedding AgPNPs within PAH/PAA BLs	5	Yes

## References

[1] Rana D., Matsuura T. (2010). Surface modifications for antifouling membranes. Chem. Rev..

[2] Saqib J., Aljundi I.H. (2016). Membrane fouling and modification using surface treatment and layer-by-layer assembly of polyelectrolytes: State-of-the-art review. J. Water Process Eng..

[3] Zhou M., Liu H., Kilduff J.E., Langer R., Anderson D.G., Belfort G. (2009). High-throughput membrane surface modification to control NOM fouling. Environ. Sci. Techn..

[4] Razmjou A., Mansouri J., Chen V. (2011). The effects of mechanical and chemical modification of TiO_2_ nanoparticles on the surface chemistry, structure and fouling performance of PES ultrafiltration membranes. J. Membr. Sci..

[5] Reddy A., Mohan D.J., Bhattacharya A., Shah V., Ghosh P. (2003). Surface modification of ultrafiltration membranes by preadsorption of a negatively charged polymer: I. Permeation of water soluble polymers and inorganic salt solutions and fouling resistance properties. J. Membr. Sci..

[6] Lv Y., Yang H.-C., Liang H.-Q., Wan L.-S., Xu Z.-K. (2015). Nanofiltration membranes via co-deposition of polydopamine/polyethylenimine followed by cross-linking. J. Membr. Sci..

[7] Kochkodan V., Hilal N. (2015). A comprehensive review on surface modified polymer membranes for biofouling mitigation. Desalination.

[8] Chung J., Chun J., Lee J., Lee S.H., Lee Y.J., Hong S.W. (2012). Sorption of Pb(II) and Cu(II) onto multi-amine grafted mesoporous silica embedded with nano-magnetite: Effects of steric factors. J. Hazard. Mater..

[9] Huang M., Chen Y., Huang C.-H., Sun P., Crittenden J. (2015). Rejection and adsorption of trace pharmaceuticals by coating a forward osmosis membrane with TiO_2_. Chem. Eng. J..

[10] Tian Y., Gao B., Morales V.L., Wang Y., Wu L. (2012). Effect of surface modification on single-walled carbon nanotube retention and transport in saturated and unsaturated porous media. J. Hazard. Mater..

[11] Ji C., Hou J., Chen V. (2016). Cross-linked carbon nanotubes-based biocatalytic membranes for micro-pollutants degradation: Performance, stability, and regeneration. J. Membr. Sci..

[12] Lee J., Chae H.-R., Won Y.J., Lee K., Lee C.-H., Lee H.H., Kim I.-C., Lee J.-M. (2013). Graphene oxide nanoplatelets composite membrane with hydrophilic and antifouling properties for wastewater treatment. J. Membr. Sci..

[13] Dong L.-X., Huang X.-C., Wang Z., Yang Z., Wang X.-M., Tang C.Y. (2016). A thin-film nanocomposite nanofiltration membrane prepared on a support with in situ embedded zeolite nanoparticles. Sep. Purif. Technol..

[14] Safarpour M., Khataee A., Vatanpour V. (2015). Thin film nanocomposite reverse osmosis membrane modified by reduced graphene oxide/TiO_2_ with improved desalination performance. J. Membr. Sci..

[15] Wang M., Wang Z., Wang X., Wang S., Ding W., Gao C. (2015). Layer-by-layer assembly of aquaporin Z-incorporated biomimetic membranes for water purification. Env. Sci. Technol..

[16] Yin J., Zhu G., Deng B. (2016). Graphene oxide (GO) enhanced polyamide (PA) thin-film nanocomposite (TFN) membrane for water purification. Desalination.

[17] Yin J., Kim E.-S., Yang J., Deng B. (2012). Fabrication of a novel thin-film nanocomposite (TFN) membrane containing MCM-41 silica nanoparticles (NPs) for water purification. J. Membr. Sci..

[18] Shi X., Shen M., Moehwald H. (2004). Polyelectrolyte multilayer nanoreactors toward the synthesis of diverse nanostructured materials. Prog. Polym. Sci..

[19] Lichter A.J., van Vliet K.J., Rubner M.F. (2009). Design of Antibacterial Surfaces and Interfaces: Polyelectrolyte Multilayers as a Multifunctional Platform. Macromolecules.

[20] DeRocher J.P., Mao P., Han J., Rubner M.F., Cohen R.E. (2010). Layer-by-Layer assembly of polyelectrolytes in nanofluidic devices. Macromolecules.

[21] Zhai L. (2013). Stimuli-responsive polymer films. Chem. Soc. Rev..

[22] Joseph N., Ahmadiannamini P., Hoogenboom R., Vankelecom I.F.J. (2014). Layer-by-layer preparation of polyelectrolyte multilayer membranes for separation. Polym. Chem..

[23] Bruening M.L., Dotzauer D.M., Jain P., Lu O., Baker G.L. (2008). Creation of functional membranes using polyelectrolyte multilayers and polymer brushes. Langmuir.

[24] Gribova V., Auzely-Velty R., Picart C. (2012). Polyelectrolyte multilayer assemblies on materials surfaces: from cell adhesion to tissue engineering. Chem. Mater..

[25] Decher G. (1997). Fuzzy nanoassemblies: toward layered polymeric multicomposites. Science.

[26] Zhai L., Berg M.C., Cebeci F.Ç., Kim Y., Milwid J.M., Rubner M.F., Cohen R.E. (2006). Patterned superhydrophobic surfaces:  toward a synthetic mimic of the Namib desert beetle. Nano Lett..

[27] Bravo J., Zhai L., Wu Z., Cohen R.E., Rubner M.F. (2007). Transparent superhydrophobic films based on silica nanoparticles. Langmuir.

[28] Kharlampieva E., Sukhishvili S.A. (2004). Competition of hydrogen-bonding and electrostatic interactions within hybrid polymer multilayers. Langmuir.

[29] Beaman K.D., Robertson E.J., Richmond G.L. (2012). Metal ions: driving the orderly assembly of polyelectrolytes at a hydrophobic surface. Langmuir.

[30] Cho C., Xiang F., Wallace K.L., Grunlan J.C. (2015). Combined ionic and hydrogen bonding in polymer multilayer thin film for high gas barrier and stretchiness. Macromolecules.

[31] Lee H. (2016). Effects of temperature, salt concentration, and the protonation state on the dynamics and hydrogen-bond interactions of polyelectrolyte multilayers on lipid membranes. Phys. Chem. Chem. Phys..

[32] Rahim M.A., Islam M.S., Bae T.S., Choi W.S., Noh Y.-Y., Lee H.-J. (2012). Metal ion-enriched polyelectrolyte complexes and their utilization in multilayer assembly and catalytic nanocomposite films. Langmuir.

[33] Mentbayeva A., Ospanova A., Tashmuhambetova Z., Sokolova V., Sukhishvili S. (2012). Polymer-metal complexes in polyelectrolyte multilayer films as catalysts for oxidation of toluene. Langmuir.

[34] Huang X., Schubert A.B., Chrisman J.D., Zacharia N.S. (2013). Formation and tunable disassembly of polyelectrolyte-Cu^2+^ layer-by-layer complex film. Langmuir.

[35] Adusumilli M. (2010). Polyelectrolyte Multilayer Films for Ion Separation and Water Purification. Ph.D. Thesis.

[36] Mallwitz F., Laschewsky A. (2005). Direct access to stable, freestanding polymer membranes by layer-by-layer assembly of polyelectrolytes. Adv. Mater..

[37] Zhai L., Cebeci F.C., Cohen R.E., Rubner M.F. (2004). Stable superhydrophobic coatings from polyelectrolyte multilayers. Nano Lett..

[38] Chunder A., Etcheverry K., Londe G., Cho H.J., Zhai L. (2009). Conformal switchable superhydrophobic/hydrophilic surfaces for microscale flow control. Colloids Surf. A Physicochem. Eng. Asp..

[39] Alpatova A., Kim E.-S., Sun X., Hwang G., Liu Y., Gamal El-Din M. (2013). Fabrication of porous polymeric nanocomposite membranes with enhanced anti-fouling properties: Effect of casting composition. J. Membr. Sci..

[40] Daraei P., Madaeni S.S., Ghaemi N., Salehi E., Khadivi M.A., Moradian R., Astinchap B. (2012). Novel polyethersulfone nanocomposite membrane prepared by PANI/Fe3O4 nanoparticles with enhanced performance for Cu(II) removal from water. J. Membr. Sci..

[41] Hoek E.M.V., Ghosh A.K., Huang X., Liong M., Zink J.I. (2011). Physical–chemical properties, separation performance, and fouling resistance of mixed-matrix ultrafiltration membranes. Desalination.

[42] Liu Y., Rosenfield E., Hu M., Mi B. (2013). Direct observation of bacterial deposition on and detachment from nanocomposite membranes embedded with silver nanoparticles. Water Res..

[43] Wu H., Tang B., Wu P. (2014). Development of novel SiO_2_–GO nanohybrid/polysulfone membrane with enhanced performance. J. Membr. Sci..

[44] Liu X., Qi S., Li Y., Yang L., Cao B., Tang C.Y. (2013). Synthesis and characterization of novel antibacterial silver nanocomposite nanofiltration and forward osmosis membranes based on layer-by-layer assembly. Water Res..

[45] Zodrow K., Brunet L., Mahendra S., Li D., Zhang A., Li Q., Alvarez P.J. (2009). Polysulfone ultrafiltration membranes impregnated with silver nanoparticles show improved biofouling resistance and virus removal. Water Res..

[46] Zhu X., Bai R., Wee K.-H., Liu C., Tang S.-L. (2010). Membrane surfaces immobilized with ionic or reduced silver and their anti-biofouling performances. J. Membr. Sci..

[47] Mohmood I., Lopes C.B., Lopes I., Ahmad I., Duarte A.C., Pereira E. (2013). Nanoscale materials and their use in water contaminants removal—A review. Environ. Sci. Pollut. Res..

[48] Pendergast M.M., Hoek E.M. (2011). A review of water treatment membrane nanotechnologies. Energy Environ. Sci..

[49] Bera A., Trivedi J.S., Kumar S.B., Chandel A.K.S., Haldar S., Jewrajka S.K. (2018). Anti-organic fouling and anti-biofouling poly(piperazineamide) thin film nanocomposite membranes for low pressure removal of heavy metal ions. J. Hazard. Mater..

[50] Firouzjaei M.D., Shamsabadi A.A., Aktij S.A., Seyedpour S.F., Sharifian Gh M., Rahimpour A., Esfahani M.R., Ulbricht M., Soroush M. (2018). Exploiting synergetic effects of graphene oxide and a silver-based metal–rganic framework to enhance antifouling and anti-biofouling properties of thin-film nanocomposite membranes. ACS Appl. Mater. Interfaces.

[51] Lin C.-F., Huang Y.-J., Hao O.J. (1999). Ultrafiltration processes for removing humic substances: Effect of molecular weight fractions and PAC treatment. Water Res..

[52] Hilal N., Ismail A.F., Wright C. (2015). Membrane Fabrication.

[53] Richardson J.J., Cui J., Björnmalm M., Braunger J.A., Ejima H., Caruso F. (2016). Innovation in layer-by-layer assembly. Chem. Rev..

[54] Prisciandaro M., di Celso G.M. (2016). On the removal of natural organic matter from superficial water by using UF and MF membranes. Desalin. Water Treat..

[55] MICRODYN-NADIR, M.-N. GmbH (2019). Trisep UA60 Product Specification.

[56] Wang C.T., Rubner M.F., Cohen R.E. (2002). Polyelectrolyte multilayer nanoreactors for preparing silver nanoparticle composites: Controlling metal concentration and nanoparticle size. Langmuir.

[57] Schuetz P., Caruso F. (2003). Copper-assisted weak polyelectrolyte multilayer formation on microspheres and subsequent film crosslinking. Adv. Funct. Mater..

[58] Sharma M., Ojha K., Ganguly A., Ganguli A.K. (2015). Ag_3_PO_4_ nanoparticle decorated on SiO_2_ spheres for efficient visible light photocatalysis. New J. Chem..

[59] Luxbacher T. (2006). Electrokinetic characterization of flat sheet membranes by streaming current measurement. Desalination.

[60] Church J. (2018). Electrochemical Microsensors for In Situ Monitoring of Chemical Compounds in Engineered and Natural Aquatic Systems. Ph.D. Thesis.

[61] Ma X., Armas S.M., Soliman M., Lytle D.A., Chumbimuni-Torres K., Tetard L., Lee W.H. (2018). In situ monitoring of Pb^2+^ leaching from the galvanic joint surface in a prepared chlorinated drinking water. Environ. Sci. Technol..

[62] Ma X., Lee W.H., Lytle D.A. (2016). In-situ 2D maps of pH shifts across brass-lead galvanic joints using microelectrodes. Meas. Sci. Technol..

[63] Mensah S.T., Gonzalez Y., Calvo-Marzal P., Chumbimuni-Torres K.Y. (2014). Nanomolar detection limits of Cd^2+^, Ag^+^, and K^+^ using paper-strip ion-selective electrodes. Anal. Chem..

[64] APHA (2017). Standard Methods for the Examination of Water and Wastewater.

[65] Nguyen N.T.X. (2017). Detection of Molecular Changes Induced by Different Classes of Antibiotics against Escherichia Coli and Vibrio Parahaemolyticus Using Raman and Infra-Red Spectroscopies. Ph.D. Thesis.

[66] Goodwin J.R. (2006). Vibrational Microspectroscopy of Bacterial Colonies. Master’s Thesis.

[67] Pryshchepa O., Sagandykova G.N., Pomastowski P., Railean-Plugaru V., Król A., Rogowska A., Rodzik A., Sprynskyy M., Buszewski B. (2019). A new approach for spontaneous silver ions immobilization onto casein. Int. J. Mol. Sci..

[68] Clay R., Cohen R. (1997). Synthesis of metal nanoclusters within microphase-separated diblock copolymers: ICP-AES analysis of metal ion uptake. Supramol. Sci..

[69] Machado G., Beppu M.M., Feil A.F., Figueroa C.A., Correia R.R.B., Teixeira S.R. (2009). Silver nanoparticles obtained in PAH/PAA-based multilayers by photochemical reaction. J. Phys. Chem. C.

[70] Joly S., Kane R., Radzilowski L., Wang T., Wu A., Cohen R., Thomas E., Rubner M. (2000). Multilayer nanoreactors for metallic and semiconducting particles. Langmuir.

[71] Jose J.A., Alagar M., Mittal V. (2015). Preparation and Characterization of Polysulfone-Based Nanocomposites. Manufacturing of Nanocomposites with Engineering Plastics.

[72] Wagner J. (2001). Membrane Filtration Handbook: Practical Tips and Hints.

[73] STERLITECH (2020). Trisep Flat Sheet Membrane UA60 PPA UF 1016 × 305 mm 1/Pk.

[74] Guerra K., Pellegrino J. (2012). Investigation of Low-Pressure Membrane Performance, Cleaning, and Economics Using a Techno-Economic Modeling Approach. Science and Technology Program Report.

[75] Park J., Park J., Kim S.H., Cho J., Bang J. (2010). Desalination membranes from pH-controlled and thermally-crosslinked layer-by-layer assembled multilayers. J. Mater. Chem..

[76] Dakal T.C., Kumar A., Majumdar R.S., Yadav V. (2016). Mechanistic basis of antimicrobial actions of silver nanoparticles. Front. Microbiol..

[77] Ahmad J., Wen X., Li F., Wang B. (2019). Novel triangular silver nanoparticle modified membranes for enhanced antifouling performance. RSC Adv..

[78] Liu C., Zhang D., He Y., Zhao X., Bai R. (2010). Modification of membrane surface for anti-biofouling performance: Effect of anti-adhesion and anti-bacteria approaches. J. Membr. Sci..

